# Proximate, mineral, and antinutrient compositions of indigenous Okra (*Abelmoschus esculentus*) pod accessions: implications for mineral bioavailability

**DOI:** 10.1002/fsn3.282

**Published:** 2015-09-17

**Authors:** Habtamu Fekadu Gemede, Gulelat Desse Haki, Fekadu Beyene, Ashagrie Z. Woldegiorgis, Sudip Kumar Rakshit

**Affiliations:** ^1^ Department of Food Technology and Process EngineeringWollega University P.O. Box 395NekemteEthiopia; ^2^ Center for Food Science and Nutrition ProgramAddis Ababa University P.O. Box 1176Addis AbabaEthiopia; ^3^ Department of Food Science and TechnologyBotswana Collage of AgricultureBotswana UniversityGaboroneBotswana; ^4^ Department of Chemical EngineeringCanada Research Chair (Tier 1)Lakehead UniversityThunder BayONCanadaP7B 5E1

**Keywords:** Accession, antinutrient, bioavailability, mineral, Okra, pod, proximate

## Abstract

The promotion and consumption of indigenous vegetables could help to mitigate food insecurity and alleviate malnutrition in developing countries. Nutrient and antinutrient compositions of eight accessions of Okra Pods were investigated. Molar ratios and mineral bioavailability of Okra pod accessions were also calculated and compared to the critical values to predict the implications for mineral bioavailability. Proximate and mineral composition of Okra pod accessions were determined using standard methods of Association of Official Analytical Chemists. The result of the study revealed that the proximate composition (g/100 g) in dry weight basis was significantly (*P* < 0.05) varied and ranged: moisture/dry matter 9.69–13.33, crude protein 10.25–26.16, crude fat 0.56–2.49, crude fiber 11.97–29.93, crude ash 5.37–11.30, utilizable carbohydrate 36.66–50.97, and gross energy 197.26–245.55 kcal/100 g. The mineral concentrations (mg/100 g) were also significantly (*P* < 0.05) varied and ranged: calcium (111.11–311.95), Iron (18.30–36.68), potassium (122.59–318.20), zinc (3.83–6.31), phosphorus (25.62–59.72), and sodium (3.33–8.31) on dry weight bases. The Okra Pods of “OPA#6” accession contained significantly higher amounts of crude protein, total ash, crude fat, calcium, iron, and zinc than all other accessions evaluated in this study. The results of antinutrients analysis showed that, except phytate, tannin, and oxalate contents of all the accessions were significantly (*P* < 0.05) varied. The range of phytate, tannin, and oxalate contents (mg/100 g) for Okra pod accessions studied were as follows: 0.83–0.87, 4.93–9.90, and 0.04–0.53, respectively. The calculated molar ratios of phytate: calcium, phytate: iron, phytate: zinc, oxalate: calcium and [Phytate][Calcium]/[Zinc] were below the critical value and this indicate that the bioavailability of calcium, iron, and zinc in these accessions could be high. The results of the study revealed that Okra pod contain appreciable amount of vital nutrients like protein, fiber, calcium, iron, and zinc and low in antinutrient contents with high mineral bioavailability. Therefore, increase in the production and consumption of these nutrient‐rich indigenous Okra pods will help to supplement/formulate the diets and alleviate the problems associated with malnutrition in the country.

## Introduction

Traditional vegetables are valuable sources of nutrients (Nesamvuni et al. 2001; Yang and Keding 2009), with some having important medicinal properties (Hilou et al. 2006). Vegetables contribute substantially to food security (Yiridoe and Anchirinah 2005). Overcoming food and nutritional insecurity among women, pregnant and lactating mothers, and children under 5 years of age, remains a challenge in many developing countries in sub‐Saharan Africa (Andersen et al. 2003; Tchientche Kamga et al. 2013).

Okra (*Abelmoschus esculentus*) is an important vegetable crop (Oyelade et al. 2003; Andras et al. 2005; Saifullah and Rabbani 2009) originated in Ethiopia (Getachew 2001; Simmone et al. 2004; Dandena 2010; Sathish and Eswar 2013). This crop is one of the most widely known and utilized species of the family Malvaceae (Naveed et al. 2009). Okra is known by many local names in different parts of the world (Nzikou et al. 2006). It is called lady's finger in England, gumbo in the United States of America, guino‐gombo in Spanish, guibeiro in Portuguese, and bhindiin India (Ndunguru and Rajabu 2004; Sorapong Benchasr 2012). In its origin of Ethiopia it is also called Kenkase (Berta), Andeha (Gumuz), Bamia (Oromica/Amharic) (Gemede et al. 2015). The name Okra probably derives from one of Niger‐Congo group of languages (the name for okra in the Twi language is nkuruma) (Benjawan et al. 2007). The term okra was in the use of English by the late 18th century (Arapitsas 2008).

Okra is a multipurpose crop due to its various uses of the pods, fresh leaves, buds, flowers, stems, and seeds. Okra immature fruits (pods), which are consumed as vegetables, can be used in salads, soups, and stews, fresh or dried, fried or boiled (Habtamu et al., 2014). Despite its nutritional compositions, Okra pod is a powerhouse of valuable nutrients (Adetuyi et al. 2011) and affordable source of protein, carbohydrates, minerals, vitamins, and dietary fiber (Habtamu et al., 2014). Therefore, promoting the consumption of Okra pods could provide cheap sources of nutrients that can improve the nutritional status and reducing the prevalence of malnutrition especially among resource‐constrained households and can also used as a means of dietary diversification. On the other hand, the presence of anti‐nutritional factors is one of the major drawbacks limiting the nutritional qualities of the food (Kathirvel and Kumudha 2011). Okra pods are not only have beneficial nutrients but might contain traces of antinutritional factors, which may have adverse effects on bioavailability of some minerals like calcium, iron, and zinc. However, okra has been considered as a minor crop and there is no single information or published studies available about nutritional, anti‐nutritional, and bioavailability of Okra pods grown in Ethiopia. Therefore, the aim of this study was to evaluate the proximate, mineral, anti‐nutrient, and bioavailability of eight indigenous Okra pod accessions grown in Benishangul Gumuz Region, Ethiopia.

## Materials and Methods

### Sample collection and preparation

Eight Okra pod accessions were harvested from Assosa agricultural research farm in Benishangul Gumiz Region, Ethiopia. Each of the collected accessions were coded, packed in polyethylene bags, kept in an ice box (to prevent moisture loss), and transported to Food Technology and Process Engineering Research laboratory of Wollega University, Ethiopia. Once in the laboratory, each of the Okra pod accessions were washed by distilled water and sliced to uniform thickness 5 mm using a stainless steel knife. The moisture content of each Okra pod accessions was determined immediate after sliced to uniform thickness. The sliced Okra Pod accessions were sun dried, followed by oven drying at 45°C. The dried Okra pod accessions were milled separately into fine powder using electric grinder until to pass through 0.425 mm sieve mesh size, and finally packed into airtight polyethylene plastic bags to minimize heat build‐up and stored in the desiccator until required for analysis.

### Proximate composition

Moisture content, total ash, crude protein, crude fiber, and crude fat of the Okra pod accessions were determined according to AOAC, (2000) using sub components 925.09, 923.03, 979.09, 962.09, and 920.39, respectively. Utilizable carbohydrate content of Okra pod accessions was calculated by difference (Manzi et al. 2004). The gross energy content of Okra pod accessions was determined by calculation from fat, carbohydrate, and protein contents using the Atwater's conversion factors; 16.7 kJ/g (4 kcal/g) for protein, 37.4 kJ/g (9 kcal/g) for fat and 16.7 kJ/g (4 kcal/g) for carbohydrates and expressed in calories (Guyot et al. 2007).

### Determination of mineral contents

Minerals content analysis was determined according to AOAC, (2000). Sodium (Na) and potassium (K) concentrations were determined by using the standard flame emission photometer; phosphorus (P) was determined colorimetrically by vanadomolybdate procedure. Calcium (Ca), iron (Fe), and zinc (Zn) concentrations were measured by atomic absorption spectrophotometer.

### Determination of antinutritional factors

Phytate was determined by the method of Vantraub and Lapteva (1988). Oxalate was analyzed using the method originally employed by Ukpabi and Ejidoh (1989) in which the procedures involve three steps: digestion, oxalate precipitation, and permanganate titration. Tannin content was determined by the method of Maxson and Rooney (1972).

### Determination of molar ratio of antinutrients to minerals

The molar ratio between antinutrient and mineral was obtained after dividing the mole of antinutrient with the mole of minerals (Woldegiorgis et al. 2015).

### Statistical analysis

The completely randomized design was used with two replicates. All the statistical analyses were performed on the results obtained using SPSS version 20.0 (SPSS Institute Inc., Cary, NC) for windows. Data were evaluated by using one way ANOVA (analysis of variance). Means of results for each experiment were separated by the Duncan's multiple range test and reported as mean ± SE (standard error). A *P*‐value of 0.05 or less was considered as statistically significant.

## Result and Discussion

### Proximate composition

Food analysis is the resolution of the components of food into its proximate or ultimate parts (Onwuka 2005). Proximate analysis involves the determination of the major components of food as moisture, ash, crude fat, crude protein, crude fiber, and carbohydrate (Ekwumemgbo et al. 2014; Aja et al. 2015). The proximate composition of Okra pod accessions is presented in Table 1.

**Table 1:** Proximate composition of eight Okra pod accessions (dry weight bases)

Accessions	Moisture content (g/100 g)	Crud protein (g/100 g)	Total ash (g/100 g)	Crude fiber (g/100 g)	Crude fat (g/100 g)	Util. carbohy. (g/100 g)	Gross energy (Kcal/100 g)
OPA#1	10.61 ± 0.27^c,d^	20.75 ± 0.52^b^	6.05 ± 0.25^c,d,e^	16.58 ± 0.05^e^	1.39 ± 0.28^b,c^	44.62 ± 0.23^b^	215.24 ± 0.56^b,c^
OPA#2	13.33 ± 0.28^a^	10.25 ± 0.69^e^	6.66 ± 0.03^c^	17.13 ± 0.39^e^	1.67 ± 0.02^b^	50.97 ± 0.00^a^	245.55 ± 0.05^a^
OPA#3	12.17 ± 0.16^b^	13.94 ± 0.02^d^	10.20 ± 0.28^b^	21.95 ± 0.03^d^	1.14 ± 0.01^c^	40.56 ± 0.49^c^	213.43 ± 0.82^c^
OPA#4	11.29 ± 0.26^c^	17.16 ± 0.65^c^	5.37 ± 0.01^e^	24.35 ± 1.17^c^	1.69 ± 0.01^b^	40.15 ± 1.55^c^	197.26 ± 2.21^d^
OPA#5	10.66 ± 0.24^c,d^	12.97 ± 0.25^d^	10.60 ± 0.17^a,b^	21.69 ± 0.19^d^	0.56 ± 0.01^d^	43.52 ± 0.85^b^	221.52 ± 2.71^b^
OPA#6	9.69 ± 0.29^e^	26.16 ± 0.12^a^	11.30 ± 0.19^a^	11.97 ± 0.83^f^	2.49 ± 0.28^a^	38.41 ± 0.56^c,d^	221.16 ± 3.9^b^
OPA#7	10.38 ± 0.26^d,e^	14.16 ± 0.14^d^	5.62 ± 0.45^d,e^	26.42 ± 0.21^b^	0.58 ± 0.01^d^	42.87 ± 1.06^b^	198.98 ± 2.41^d^
OPA#8	10.22 ± 0.22^d,e^	16.24 ± 0.94^c^	6.39 ± 49^c,d^	29.93 ± 0.09^a^	0.56 ± 0.01^d^	36.66 ± 0.85^d^	214.16 ± 1.38^e^

Means not followed by the same superscript letters in the same column are significantly different (*P* < 0.05).

Data are expressed as mean ± SE of replicate determinations (*n* = 2).

OPA stands for Okra Pod Accession and # stands for number.

Moisture content determination is an integral part of the proximate composition analysis of food. Moisture content of eight accessions of Okra pods is presented in Table 1. As fresh Okra pods vary considerably in water content, moisture contents were calculated on a dry weight basis, which allows a greater consistency of data. This implies a factor from 1.10 to 1.14 must be multiplied as moisture correction factor for all the other analysis parameters. Okra pod accession “OPA#2” had the highest moisture/dry matter content (13.33 g/100 g) which was significantly (*P* < 0.05) higher than the moisture/dry matter content of all the accessions whereas Okra pod accession “OPA#6” had significantly (*P* < 0.05) lower than the moisture/dry matter content (9.69 g/100 g) of all the accessions except accession “OPA#8” (10.22 g/100 g) and “OPA#7” (10.38 g/100 g). Although fresh Okra pod accessions were ranged from 87.98 to 90.60 g/100 g water and this indicate that the Okra pods have a high moisture content. The high moisture content in okra pods accessions is in agreement with the finding of Adetuyi et al. (2011). Also this is in accordance with the finding of Gopalan et al. (2007) (89 g/100 g) and (Nwachukwu et al. 2014) (88.47 g/100 g). Moisture content of any food is an index of its water activity and is used as a measure of stability and susceptibility to microbial contamination (Uyoh et al. 2013). The high moisture content in vegetables makes them vulnerable to microbial attack, hence spoilage (Nwofia et al., 2012). This high moisture content also implies that dehydration would increase the relative concentrations of other food nutrient and therefore improve the shelf‐life and preservation of the fruits (Aruah et al. 2012). There is also need to store the fruit in cool condition if they are to be kept for a long period without spoilage especially in the tropics were wastage of vegetable crops is estimated to be around 50% due to high moisture content (Nwofia, 2012).

The main functions of proteins are growth and replacement of lost tissues in the human body. Table 1 shows the crude protein contents of the eight accessions of Okra pod used in the study. The protein content of the Okra pod accessions was varied significantly (*P* < 0.05) from 10.25 g/100 g in “OPA#2” to 26.16 g/100 g in “OPA#6” on dry weight basis and this variation might be due to genetic factor. The mean value of the accessions obtained in the study is almost comparable with the find of Adetuya et al. (2011) (13.61–16.27 g/100 g) while higher than the value reported by Nwachukwu et al. (2014) (4.81 g/100 g). Ogungbenle and Omosola (2015) is also reported that the crude protein content of Okra pod is (23.4 g/100 g) which is higher than all the accession in the present study except “OPA#6” (26.16 g/100 g). Okra can be considered a high protein vegetable when compared with *moringa oliefera* (4.2 g/100 g), *Amarantus* (6.1 g/100 g)*, Gnetum Africanum* (1.5 g/100 g), and *Pterocarpus* (2.0 g/100 g) (Nzikou et al. 2006) and this implies that Okra pod can serve as a good source of protein. Nwofia et al. (2012) reported that diet is nutritionally satisfactory, if it contains high caloric value and a sufficient amount of protein. It have been shown that any plant foods that provides about 12% of their calorific value from protein are considered good source of protein(Effiong et al. 2009; Ali 2010). The pods of these accessions of Okra meet this requirements and this implies that Okra pod can serve as a good source of protein.

Crude fat content of eight accessions of Okra pods is presented in Table 1. The levels of crude fat varied from 0.56 g/100 g (“OPA #8” and “OPA #5”) to 2.49 g/100 g (“OPA #6”). Okra pod accession “OPA#6” had the highest crude fat content (2.49 g/100 g) which was significantly (*P* < 0.05) higher than the crude fat content of all the accessions whereas Okra pod accession “OPA#8” and “OPA#5” had significantly (*P* < 0.05) lower than the crude fat content (0.56 g/100 g) of all the accessions except accession “OPA#7” (0.58 g/100 g). In these finding, the crude fat content is higher than the value reported by Nwachukwu et al. (2014) (0.18 g/100 g) whereas lower than the value reported by Adetuyi et al. (2011) (9.22–10.57 g/100 g). Dietary fats function in the increase of palatability of food by absorbing and retaining flavors (Antia et al. 2006). Excess consumption of fat have been implicated in certain cardiovascular disorders such as atherosclerosis, cancer, and aging, whereas a diet providing 1–2% of its caloric of energy as fat is said to be sufficient to human beings (Aruah et al. 2011), in this regard, the consumption of Okra pod diet should be encouraged to reduce the risk of above diseases in man.

Dietary fiber promotes the growth and protects the beneficial intestinal flora. Moreover, high intake of fiber reduces the risk of colon cancer (Dawczynski et al. 2007). The crude fiber content among the accessions of Okra pod varied from 11.97 g/100 g to 29.93 g/100 g in “OPA#6” and “OPA#8” accessions, respectively. The accession, “OPA#8” had significantly (*P* < 0.05) higher (29.93 g/100 g) in crude fiber content and was followed by “OPA#7” (26.42 g/100 g), “OPA#4” (24.35 g/100 g), “OPA#3” (21.95 g/100 g) in that order, however, the accession “OPA#6” recorded the lowest (11.97 g/100 g) crude fiber content on dry weight basis. Adetuya et al. (2011) reported that the fiber content of Okra pod ranges from 10.15 to 11.63 g/100 g which is lower than the crude fiber obtained in this study. Adetuya et al. (2011) also reported that the fiber content of okra is high when compared with *Amarantus hybridus* (1.6 g/100 g) and *Laurea taraxifolia* (2.0 g/100 g) but very low in comparison with *Gnetumn africanum* (3.0 g/100 g). In this study, the fiber content is relatively high which may suggest that consumption of okra will can improve its digestibility and absorption processes in large intestine, helping to stimulate peristalsis, thereby preventing constipation (Ogungbenle and Omosola 2015). Interest in fiber evaluation has increased due to the recent information on the potential role of dietary fiber in human nutrition (Mensan et al. 2008). Evidences from epidemiological studies suggest that high fiber consumption may contribute to a reduction in the incidence of certain diseases like diabetes, coronary heart disease, colon cancer, high blood pressure, obesity, and various digestive disorders (Ikewuchi and Ikewuchi 2009). Dietary fiber is known to alter the coronary environment in such a way as to protect against colorectal diseases (Ekumankama 2008). It provides protection by increasing fecal bulk, which dilutes the increased colonic bile that occurs with high fat diet (Dillard and German 2000; Zhao et al. 2007). When found in excess, it may bind some essential trace elements leading to deficiency of some minerals such as iron and zinc (Ekwumemgbo et al. 2014). Therefore, okra pod diet is considered as a main source of crude fiber.

The ash content is a measure/reflection of the nutritionally important mineral contents present in the food material (Omotosho 2005; Nnamani et al. 2009). Table 1 shows the crude ash contents of the eight accessions of Okra pod used in the study. The level of ash content was ranged from 5.37 g/100 g (OPA#4) to 11.30 g/100 g (OPA#6). The ash content was significantly (*P* < 0.05) higher in “OPA#6” (11.30 g/100 g) followed by “OPA#3” (10.20 g/100 g), “OPA#2” (6.66 g/100 g), and “OPA#8” (6.39 g/100 g) in that order, whereas accession “OPA#4” had the lowest (5.37 g/100 g) ash content value but this did not differ significantly (*P* < 0.05) from accession “OPA#7” (5.62 g/100 g) and “OPA#1” (6.05 g/100 g) on dry weight basis. The results showed that the sample contains high ash content which indicates that the okra pods would provide essential valuable and useful minerals needed for body development. The mean of ash content in this result agrees with the findings of Adetuya et al. (2011) (7.19–9.63 g/100 g).

Table 1 shows the utilizable carbohydrate contents of the eight accessions of Okra pod used in the study. The utilizable carbohydrate content of Okra pod accessions varied from 36.66 g/100 g to 50.97 g/100 g in “OPA#8” and “OPA#2” accessions, respectively. Utilizable carbohydrate content of pod accession “OPA#2” had higher (50.97 g/100 g), whereas accession “OPA#8” had the lowest (36.66 g/100 g) but this did not differ significantly from accession “OPA#6” (38.41 g/100 g) utilizable carbohydrate contents. The sample could be considered as potential source of carbohydrate when compared with the content of some conventional sources like cereals with 72–90 g/100 g Carbohydrate (Elinge et al. 2012).

The gross energy was calculated by multiplying the mean values of crude proteins, crude fat, and total carbohydrate by Atwater factors of 4, 9, and 4, respectively. Gross energy content was ranged from 197.26 kcal/100 g in “OPA#4” to 245.55 kcal/100 g in “OPA#2” accessions. Gross energy content of pod accession “OPA#2” had higher (245.55 kcal/100 g), whereas accession “OPA#4” had the lowest (197.26 kcal/100 g) but this did not differ significantly (*P* < 0.05) from accession “OPA#7” (198.98 kcal/100 g) on dry weight basis. This energy value indicates that these accessions can serve as a good source of energy for the body.

### Mineral composition

Minerals are considered to be essential in human nutrition (Ibanga and Okon 2009; Valvi and Rathod 2011). These minerals are vital for the overall mental and physical well being and are important constituents of bones, teeth, tissues, muscles, blood, and nerve cells (Soetan et al. 2010). They also help in the maintenance of acid‐base balance, response of nerves to physiological stimulation and blood clotting (Hanif et al. 2006). The mineral composition of eight Okra pod accessions is shown in Table 2.

**Table 2:** Mineral concentrations of eight Okra pod accessions (dry weight bases)

Accessions	Mineral content (mg/100 g)					
	Calcium	Iron	Potassium	Zinc	Phosphorous	Sodium
OPA#1	111.11 ± 0.37^g^	25.41 ± 1.36^c,d^	169.82 ± 8.25^c^	4.13 ± 0.04^d,e^	33.02 ± 0.46^e^	5.01 ± 0.54^b,c^
OPA#2	276.29 ± 0.96^b^	20.98 ± 0.75^d,e^	318.20 ± 6.67^a^	4.61 ± 0.01^c^	42.17 ± 0.78^c^	8.31 ± 0.51^a^
OPA#3	311.95 ± 0.57^a^	31.77 ± 0.37^b^	177.96 ± 2.89^c^	6.30 ± 0.09^a^	27.61 ± 0.91^f^	3.97 ± 0.56^b,c^
OPA#4	140.88 ± 1.39^f^	23.30 ± 0.48^d^	277.82 ± 9.62^b^	4.16 ± 0.09^c,d,e^	54.11 ± 1.62^b^	5.61 ± 1.13^b,c^
OPA#5	253.52 ± 4.02^c^	36.68 ± 0.84^a^	122.59 ± 11.00^d^	3.83 ± 0.24^e^	59.72 ± 0.55^a^	3.91 ± 0.57^b,c^
OPA#6	311.35 ± 0.27^a^	32.90 ± 2.65^a,b^	263.12 ± 1.06^b^	6.31 ± 0.19^a^	36.32 ± 0.68^d^	6.06 ± 0.57^a,b^
OPA#7	188.79 ± 3.30^e^	18.30 ± 0.18^e^	183.52 ± 7.79^c^	5.65 ± 0.05^b^	25.62 ± 0.83^f^	4.99 ± 0.57^b,c^
OPA#8	203.89 ± 1.08^d^	28.49 ± 1.77^b,c^	174.04 ± 2.75^c^	4.35 ± 0.19^c,d^	58.48 ± 1.21^a^	3.33 ± 1.11^c^

Means not followed by the same superscript letters in the same column are significantly different (*P* < 0.05).

Data are expressed as mean ± SE of replicate determinations (*n* = 2).

OPA stands for Okra Pod Accession and # stands for number.

Calcium is the major component of bone and assists in teeth development. Calcium concentrations are also necessary for blood coagulation and for the integrity of intracellular cement substances (Okaka and Okaka 2001). Calcium content in the eight Okra pod accessions is shown in Table 2. The concentration of Calcium in the sample is varied from 111.11 mg/100 g to 311.95 mg/100 g in “OPA#1” and “OPA#3” accessions, respectively. Okra pod accession “OPA#3” had the highest calcium content (311.95 mg/100 g) which was significantly (*P* < 0.05) higher than the Calcium content of all the accessions except accession “OPA#6” (311.35 mg/100 g), whereas Okra pod accession “OPA#1” had the lowest Calcium content (11.11 mg/100 g) on dry weight basis. This result appeared to be far higher than the Calcium contents of Okra variety reported by Adetuya et al. (2011) varying between 58.22 mg/100 g to 58.31 mg/100 g.

Iron is an essential trace element for hemoglobin format on, normal functioning of central nervous system and in the oxidation of carbohydrates, protein, and fats (Kermanshah et al. 2014; Mlitan et al. 2014). It also facilitates carbohydrates, protein, and fat to control body weight, which is very important factor in diabetes (Moses et al. 2012). Iron is necessary for the formation of hemoglobin and also plays an important role in oxygen transfer in human body and low iron content causes gastrointestinal infection, nose bleeding myocardial infection (Ullah et al. 2012). Table 2 shows Iron content of the eight accessions of Okra pod used in the study. The contents of Iron varied from 18.30 mg/100 g in “OPA#7” to 36.68 mg/100 g in “OPA#5”. The Iron content of Okra pod accession “OPA#5” had higher (36.68 mg/100 g), but this did not differ significantly (*P* < 0.05) from accession “OPA#6” (32.90 mg/100 g), whereas accession “OPA#7” had the lowest (18.30 mg/100 g) but nonsignificant (*P* < 0.05) from accession “OPA#2” (20.98 mg/100 g) on dry weight basis. The values obtained in this study were far higher than the value reported by Adetuya et al. (2011) which is varied from 0.87 mg/100 g to 0.96 mg/100 g. This indicates that Okra pod is a rich source of Iron.

Zinc is an essential trace element and plays an important role in various cell processes including normal growth, brain development, behavioral response, bone formation, and wound healing (Mlitan et al. 2014). Zinc also plays a very important role in protein and carbohydrate metabolism and also help in mobilizing vitamin A from its storage site in the liver and facilitates the synthesis of DNA and RNA necessary for cell production (Jabeen et al. 2010). Zinc deficiency is common in people suffering from Chrohn's disease, hypothyroidism, and gum disease, and probably plays a part in susceptibility to viral infections and diabetes mellitus. It can be beneficial in the treatment of viral infections, including those of AIDS, prostate gland enlargement, rheumatoid arthritis, healing of wounds, acne, eczema, and stress (Kermanshah et al. 2014). Zinc content in the eight Okra pod accessions is shown in Table 2. The content of Zinc varied between 3.83 mg/100 g in “OPA#5” and 6.31 mg/100 g in “OPA#6”. Zinc content of pod accession “OPA#6” had higher (6.31 mg/100 g) but this did not differ significantly from accession “OPA#3” (6.30 mg/100 g) while accession “OPA#5” had the lowest (3.83 mg/100 g) but this did not differ significantly (*P* < 0.05) from Okra pod accession “OPA#1” (4.13 mg/100 g) and “OPA#4” (4.16 mg/100 g) on dry weight basis. The values obtained in this study are higher than the values reported by Adetuya et al., (2011) (1.29 mg/100 g–1.37 mg/100 g).

Phosphorus content of the accessions of eight Okra pod is shown in Table 2. In this study, the phosphorus content is varied from 25.62 mg/100 g (OPA#7) to 59.72 mg/100 g (OPA#5). The Phosphorus content of pod accession “OPA#5” had higher (59.72 mg/100 g) but did not differ significantly from accession “OPA#8” (58.48 mg/100 g) while accession “OPA#7” had the lowest (25.62 mg/100 g) but did not differ significantly (*P* < 0.05) from Okra pod accession “OPA#3” (27.61 mg/100 g) on dry weight basis. The value of this study is lower than the value reported by Adetuya et al. (2011) which is varied from 60.05 mg/100 g to 62.17 mg/100 g.

Potassium content of the accessions of eight Okra pod is shown in Table 2. The Potassium content of pod accession “OPA#2” had significantly (*P* < 0.05) higher (318.20 mg/100 g), whereas accession “OPA#5” had the lowest (122.59 mg/100 g) on dry weight basis. High amount of potassium in the body was reported to increase iron utilization (Elinge et al. 2012) and beneficial to people taking diuretics to control hypertension and suffer from excessive excretion of potassium through the body fluid (Arinanthan et al. 2003).

Sodium content of the accessions of eight Okra pod is shown in Table 2. In this study, the sodium content is varied from 3.33 mg/100 g (OPA#8) to 8.31 mg/100 g (OPA#2). Sodium content of Okra pod accession “OPA#2” had higher (8.31 mg/100 g) but this did not differ significantly from accession “OPA#6” (6.06 mg/100 g) while accession “OPA#8” had the lowest (3.33 mg/100 g) but this did not differ significantly (*P* < 0.05) from all the five remaining accessions on dry weight basis.

### Antinutritional factors

Antinutritional factors are a chemical compounds synthesized in natural food and/or feedstuffs by the normal metabolism of species which exerts effect contrary to optimum nutrition (Gemede and Ratta 2014). Antinutritional factors are also reduce the maximum utilization of nutrients especially proteins, vitamins, and minerals, thus preventing optimal exploitation of the nutrients present in a food and decreasing the nutritive value (Ugwu and Oranye 2006; Fekadu et al. 2013). The antinutritional composition of eight Okra pods accessions are shown in Table 3.

**Table 3:** Antinutritional factors content of the accessions of eight Okra pod (dry weight bases)

Accessions	Phytate (mg/100 g)	Oxalate (mg/100 g)	Tannin (mg/100 g)
OPA#1	0.85 ± 0.01^a^	0.04 ± 0.04^e^	7.61 ± 0.55^b,c^
OPA#2	0.85 ± 0.01^a^	0.53 ± 0.53^a^	6.75 ± 0.32^c,d^
OPA#3	0.87 ± 0.02^a^	0.09 ± 0.09^c,d^	5.75 ± 0.38^d,e^
OPA#4	0.83 ± 0.02^a^	0.15 ± 0.15^c^	8.12 ± 0.38^b^
OPA#5	0.83 ± 0.01^a^	0.06 ± 0.06^e^	7.48 ± 0.33^b,c^
OPA#6	0.85 ± 0.02^a^	0.28 ± 0.28^b^	9.70 ± 0.41^a^
OPA#7	0.84 ± 0.01^a^	0.47 ± 0.47^a^	4.93 ± 0.15^e^
OPA#8	0.86 ± 0.03^a^	0.12 ± 0.12^c,d^	9.90 ± 0.46^a^

Means not followed by the same superscript letters in the same column are significantly different (*P* < 0.05).

Data are expressed as mean ± SE of replicate determinations (*n* = 2).

OPA stands for Okra Pod Accession and # stands for number.

The phytate content of Okra pod accession was highest in “OPA#3” (0.87 mg/100 g) and lowest in “OPA#5” and “OPA#4” (0.83 mg/100 g) whereas value of Okra pod accession “OPA#3” was nonsignificant (*P* < 0.05) from phytate content of the remaining accessions on dry weight bases. The problem with phytate in food is that it can bind some essential mineral nutrients in the digestive tract and can result in mineral deficiencies (Bello et al. 2008). The phytate composition of the sample might not pose any health hazard when compared with a phytate diet of 10–60 mg/100 g which if consumed over a long period of time that has been reported to decrease bioavailability of minerals (Elinge et al. 2012). On the other hand, currently there is evidence that dietary phytate at low level may have beneficial role as an antioxidant, anticarcinogens and likely play an important role in controlling hypercholesterolemia and atherosclerosis (Phillippy et al. 2004). The result of this study is lower than the value reported by Adetuya et al., (2011) (2.64–3.90 mg/100 g). Because Okra pod may provide a substantial portion of phytate, the health benefits of phytate in Okra pod should be investigated.

Tannin content of pod accession “OPA#8” (9.90 mg/100 g) had higher but did not differ significantly from accession “OPA#6” (9.70 mg/100 g) while accession “OPA#7” (4.93 mg/100 g) had the lowest but did not differ significantly (*P* < 0.05) from Okra pod accession “OPA#3” (5.74 mg/100 g) on dry weight basis. Tannins had been reported to affect protein digestibility, adversely influencing the bioavailability of nonhem iron leading to poor iron and calcium absorption, also carbohydrate is affected leading to reduced energy value of a diet containing tannins (Adeparusi 2001), however, its antinutritional/toxicity effects depend upon their chemical structure and dosage (Fekadu et al. 2013). Therefore, the toxicity effects of the tannin may not be significant since the total acceptable tannic acid daily intake for a man is 560 mg/100 g (Fekadu et al. 2013). Since the tannin content of Okra pod accessions are very low compared to its critical toxicity effect and further reduced during traditional processing, its antinutritional effect may be insignificant in both raw and processed Okra pod.

Oxalate content of pod accession “OPA#2” (0.53 mg/100 g) had higher but this did not differ significantly (*P* < 0.05) from accession “OPA#7” (0.47 mg/100 g) while accession “OPA#1” (0.04 mg/100 g) had the lowest but did not differ significantly (*P* < 0.05) from Okra pod accession “OPA#5” (0.06 mg/100 g), “OPA#3” (0.09 mg/100 g) and “OPA#8” (0.12 mg/100 g) on dry weight basis. The result of this study is comparable with the finding of Adetuya et al., (2011) (0.32–0.506 mg/100 g). Oxalates can have a harmful effect on human nutrition and health, especially by reducing calcium absorption and aiding the formation of kidney stones (Fekadu et al. 2013). High‐oxalate diets can increase the risk of renal calcium oxalate formation in certain groups of people (Gemede and Ratta 2014). The majority of urinary stones formed in humans are calcium oxalate stones and currently, patients are advised to limit their intake of foods with a total intake of oxalate not exceeding 50–60 mg per day (Massey et al. 2001). The traditionally processed Anchote tubers analyzed in this study are low compared to the recommendations for patients with calcium oxalate kidney stones. Under these guidelines, processed Anchote tubers analyzed could be recommended not only for normal healthy people but also consumption for patients with a history of calcium oxalate kidney stones, assume about 1 kg of Anchote would be necessary for consumption per day.

### Molar ratios and bioavailability of minerals

The molar ratios for oxalate, calcium, zinc, iron, and phytate were calculated to evaluate the effects of elevated levels of oxalate and phytate in the bioavailability of dietary minerals. Bioavailability is the ability of the body to digest and absorb the mineral in the food consumed (Fekadu et al. 2013). The calculated values are also compared with the reported critical toxicity values for these ratios. The calculated Phy:Ca, Ox: Ca, Phy: Zn, Phy: Fe, and [Ca] [Phy]/[Zn] molar ratios of Okra pod accessions are shown in Table 4.

**Table 4:** Calculated molar ratio Phy:Ca Phy:Fe, Phy:Zn, Ox:Ca, and [Ca][Phy]/[Zn] molar ratios of Okra pod accessions

Accessions	Phytate: Ca (Molar ratio)	Phytate: Fe (Molar ratio)	Phytate: Zn (Molar ratio)	Oxalate: Ca (Molar ratio)	Phytate*Ca: Zn (mol/kg)
OPA#1	0.0047 ± 0.050^a^	0.0028 ± 0.012^c,d^	0.0204 ± 0.044^a,b^	0.0016 ± 0.0405^e,f^	0.0565 ± 0.0014^c^
OPA#2	0.0019 ± 0.007^d,e^	0.0034 ± 0.010^b^	0.0182 ± 0.013^b^	0.0087 ± 0.0775^b^	0.1252 ± 0.0013^a^
OPA#3	0.0017 ± 0.050^e^	0.0023 ± 0.001^e,f^	0.0136 ± 0.038^c^	0.0013 ± 0.0145^e,f^	0.1059 ± 0.0001^b^
OPA#4	0.0036 ± 0.170^b^	0.0030 ± 0.001^b,c^	0.0198 ± 0.092^a,b^	0.0049 ± 0.0695^c^	0.0694 ± 0.0025^c^
OPA#5	0.0010 ± 0.078^d^	0.0019 ± 0.007^f^	0.0216 ± 0.016^a^	0.0010 ± 0.0255^f^	0.1363 ± 0.0079^a^
OPA#6	0.0017 ± 0.057^e^	0.0022 ± 0.023^f^	0.0134 ± 0.087^c^	0.0040 ± 0.0220^c,d^	0.1037 ± 0.0008^b^
OPA#7	0.0027 ± 0.021^c^	0.0039 ± 0.008^a^	0.0147 ± 0.004^c^	0.0011 ± 0.0525^a^	0.0693 ± 0.0014^c^
OPA#8	0.0026 ± 0.120^c^	0.0026 ± 0.023^d,e^	0.0195 ± 0.014^a,b^	0.0027 ± 0.0460^d,e^	0.0994 ± 0.0066^b^

Means not followed by the same superscript letters in the same column are significantly different (*P* < 0.05).

Phytic acids markedly decrease Ca bioavailability and the Phy: Ca molar ratio has been proposed as an indicator of Ca bioavailability. The critical molar ratio of [phy]: [Ca] of < 0.24 indicating good calcium bioavailability (Woldegiorgis et al. 2015). The values in this study were lower in all accessions than the reported critical molar ratio of Phytate to Calcium, indicating that absorption of calcium not adversely affected by phytate in all the accessions.

Phytate begins to lose its inhibitory effect on iron absorption when phytate:iron molar ratios are less than 1.0, although even ratios as low as 0.2 exert some negative effect (Hurrell et al., 2003). The phytate:iron molar ratios greater than 0.15 regarded as indicative of poor iron bioavailability (Siegenberg et al. 1991). This result indicated that, the phytate:iron molar ratios of all the accessions are less than the critical value, which implies the absorption of iron all the accessions not inhibited by phytate and as a result the bioavailability of iron is good.

The importance of foodstuffs as a source of dietary zinc depends on both the total zinc content and the level of other constituents in the diet that affect zinc bioavailability. Phytate may reduce the bioavailability of dietary zinc by forming insoluble mineral chelates at a physiological pH (Bhandari and Kawabata 2004) and the formation of the chelates depends on relative levels of both zinc and phytic acid. Hence, the phytate: Zn molar ratio is considered a better indicator of zinc bioavailability than total dietary phytate levels alone (Woldegiorgis et al. 2014). Therefore, the foods with a molar ratio of Phy: Zn less than 10 showed adequate availability of Zn and problems were encountered when the value was >15. Phytate: zinc molar ratios >15, indicative of poor zinc bioavailability (Morris and Ellis 1989). The values of Okra pod accessions were lower than the critical molar ratios of Phy:Zn, which indicates the availability of zinc good.

Oxalic acid and its salts can have deleterious effects on human nutrition and health, particularly by decreasing calcium absorption and aiding the formation of kidney stones (Bhandari and Kawabata 2004). The importance of oxalate contents of an individual plant product in limiting total dietary Ca availability is of significance only when the ratio of Ox:Ca is greater than one (Frontela et al. 2009). From the result, it was observed that, all Okra pod accessions had Ox:Ca values are lower than the reported critical value (1.0), which implies that a low level of oxalate could have no adverse effects on bioavailability of dietary calcium in these accessions.

The potentiating effect of calcium on zinc absorption in the presence of high phytate intakes has led to the suggestion that the [Phy][Ca]/[Zn] millimolar ratio may be a better index of zinc bioavailability than the [Phy]/[Zn] molar ratio alone (Obah and Amusan 2009). High calcium levels in foods can promote the phytate‐induced decrease in zinc bioavailability when the [Ca][phytate]/[Zn] millimolar ratio exceeds 0.5 mol/kg (Adetuyi et al. 2011). In this study, the values of [Ca][Phy]/[Zn] millimolar ratios of all the accessions were found less than the critical level.

## Conclusions

In conclusion, the study revealed that there is a significant difference (*P* < 0.05) in the proximate and mineral compositions of Okra pod accessions. The most remarkable finding of this study is that Okra pod accessions were found to be a good source of vital nutrients like crude protein, crude fiber, crude ash, calcium, and iron. Specifically, Okra pod of “OPA#6” accession contained significantly higher amounts of crude protein, total ash, crude fat, gross energy, calcium, iron, and zinc than all other accessions and can be recommended as a remedy to alleviate malnutrition in the country. Interestingly, the antinutritional contents of the Okra pods were low and the bioavailability of calcium, iron, and zinc were high and therefore, its cultivation and consumption is encouraged as additional source of minerals to the diet of the indigenous people. Therefore, Okra pods could be employed in fortification, formulation and supplementation of other food materials.

## Conflict of Interest

None declared.
